# Current Diagnosis and Treatment of Anti-Neutrophil Cytoplasmic Antibody-Associated Vasculitis: A Review Including a Comparison of Characteristics in Europe and Japan

**DOI:** 10.3390/jcm14051724

**Published:** 2025-03-04

**Authors:** Yoshiro Horai, Shota Kurushima, Atsushi Kawakami

**Affiliations:** 1 Department of Rheumatology, Sasebo City General Hospital, Sasebo 857-8511, Japan; 2 Department of Immunology and Rheumatology, Division of Advanced Preventive Medical Sciences, Nagasaki University Graduate School of Biomedical Sciences, Nagasaki 852-8523, Japan

**Keywords:** anti-neutrophil cytoplasmic antibody-associated vasculitis, avacopan, cyclophosphamide, eosinophilic granulomatosis with polyangiitis, glucocorticoid, granulomatous polyangiitis, microscopic polyangiitis, rituximab

## Abstract

Anti-neutrophil cytoplasmic antibody (ANCA)-associated vasculitis (AAV) is a disease entity characterized by systemic vasculitis positive for ANCAs, which often leads to severe organ damage such as diffuse bronchoalveolar hemorrhage and rapidly progressive glomerulonephritis. It is known that the incidence and characteristics of AAV vary depending on region, and differences in the peak age of onset, the ratio of positive rates of MPO-ANCA to PR3-ANCA, and occurrence rates of GPA and MPA may have resulted in different approaches to clinical practice. It may also be necessary to modify therapeutic strategies according to ethnic factors. Avacopan is a therapeutic option recently recommended for the management of AAV; however, the rate of severe liver injuries associated with avacopan was reported to be relatively high in the Japanese population. In this review, we introduce current globally recognized knowledge on the diagnosis and treatment of AAV, including a comparison of patient characteristics and clinical practice between Europe and Japan obtained from the recent literature.

## 1. Introduction

Anti-neutrophil cytoplasmic antibody (ANCA)-associated vasculitis (AAV), which is classified as granulomatous with polyangiitis (GPA), microscopic polyangiitis (MPA), or eosinophilic granulomatosis with polyangiitis (EGPA), is a disease entity characterized by systemic vasculitis positive for ANCAs. AAV often leads to severe organ damage such as diffuse bronchoalveolar hemorrhage (DAH) and glomerulonephritis (GN), which often takes the form of rapidly progressive GN (RPGN), which necessitates intensive care unit treatment and/or is fatal at a certain point [1]. Patients with AAV might be encountered by ophthalmologists [2], otorhinolaryngologists [3], or dentists [4], especially in cases where symptoms are limited to head and neck lesions, before visceral damage becomes prominent. Therefore, the importance of the early diagnosis of AAV should be acknowledged not only by pulmonologists, nephrologists, and rheumatologists but also by specialists in other areas.

It is known that the incidence of AAV varies depending on the region: GPA is more common in Northern Europe, whereas occurrence rates of MPA are relatively high in Southern Europe, China, and Japan [5,6]. In addition, the clinical characteristics of AAV in the Japanese population are different to those in the European population [7,8]: MPA is relatively common compared to GPA and has a later onset compared to European populations, which suggests the necessity of a different therapeutic approach for Japanese patients [9,10].

Although glucocorticoids (GCs) were the initial cornerstone therapy against AAV, the cumulative toxicity of GCs is not negligible. The introduction of B-cell-depleting immunosuppressants, such as cyclophosphamide (CY) [11] and rituximab (RTX), has improved the prognosis of patients with AAV [12,13,14]; however, even these immunosuppressants have possible adverse effects, such as opportunistic infections, which might affect the prognosis of patients with AAV. In recent years, a newly introduced C5a receptor inhibitor, avacopan, the efficacy of which was analyzed in a phase 3 randomized controlled trial named the ADVOCATE study, showed potential as a replacement for GCs in the treatment of GPA and MPA, which would contribute to reducing the toxicity of GCs [15]. However, despite the entry criteria set in the ADVOCATE study, such as the required concomitant use of CY/RTX and the exclusion of patients with severe renal dysfunction, whether the benefits of avacopan can be observed in patients in whom CY and RTX cannot be used or who have a severe form of AAV, such as RPGN, remains to be determined. Furthermore, cases of severe liver dysfunction associated with avacopan have been reported in Japan [16,17,18,19,20]. The appropriate use of avacopan, including avoiding the liver injury often found in Japanese patients, remains to be studied in actual clinical practice.

In this article, we discuss current issues in AAV, ranging from precise diagnosis and assessment to treatment, including recently introduced knowledge on avacopan. We identified English publications of original research and clinical practice recommendations/guidelines on AAV by searching PubMed. As described above, the characteristics of Japanese patients with AAV differ from those of European patients in several aspects [7,8,9,10]. We introduce current knowledge on the clinical treatment of GPA and MPA characteristic of the Japanese population, comparing it to that of European countries. To the best of our knowledge, this is the first review to compare clinical practice between Europe and Japan based on the current literature. Among the different forms of AAV, EGPA, in which the positive rate of ANCA is relatively low compared to the other two forms of AAV [21,22], is thought to be a distinct disease entity requiring a different approach to diagnosis and treatment [23]. Considering this, we mainly focus on GPA and MPA in Section 3, which pertains to treatment. Anti-interleukin 5 therapies such as mepolizumab and benralizumab have been revolutionizing clinical practice in EGPA; therefore, we also discuss current therapies for EGPA, including these biological drugs, in Section 3.

## 2. Classification, Biomarkers, and Accompanying Rheumatic Diseases of AAV

The flowchart classifying vasculitis into GPA, MPA, EGPA, and polyarteritis nodosa, proposed by Watts et al. [24], has been utilized for a long time. In 2022, the American College of Rheumatology (ACR) and European Alliance of Associations for Rheumatology (EULAR) collaboratively announced classification criteria for GPA [25], MPA [26], and EGPA [27]. In Japan, the criteria for Wegener’s granulomatosis, MPA, and allergic granulomatous angiitis proposed by the Japan Research Committee of the Ministry of Health, Labour, and Welfare (JMHLW) [28] are still adopted for the diagnosis of diseases designated intractable by the Japanese government, with the name ‘Wegener’s granulomatosis’ replaced by GPA and ‘allergic granulomatous angiitis’ replaced by EGPA. In addition, in the JMHLW criteria, each form of AAV is classified as ‘definite’ or ‘probable’ depending on the degree of compatibility with each criteria. Consistencies between Watts’ algorithm and the JMHLW criteria, Watts’ algorithm and the 2022 ACR/EULAR criteria, and the 2022 ACR/EULAR criteria and the JMHLW criteria in the Japanese population have previously been examined; there was a difference between Watts’ algorithm and the JMHLW criteria with regard to whether ‘definite’ and ‘probable’ were included or excluded [29]. When comparing Watts’ algorithm and the 2022 ACR/EULAR criteria, the 2022 ACR/EULAR criteria were found to better classify each form of AAV [30]. The results of classification based on the JMHLW criteria, with the condition that ‘probable’ cases were included, were relatively compatible with those of the 2022 ACR/EULAR criteria [31].

The lungs and kidneys are the main organs affected in GPA and MPA. Interstitial lung disease (ILD), which usually emerges before a patient is classified as having AAV [32], is common in MPO-ANCA-positive AAV that manifests as various radiographic patterns, usually interstitial pneumonia, non-specific interstitial pneumonia, or bronchiolitis [33]. Patients with MPA in Japan were more frequently positive for MPO-ANCA and interstitial pneumonia compared to those in Europe [9]. DAH is a severe, life-threatening form of lung involvement in AAV. In a retrospective, multicenter study of MPO-ANCA-positive MPA patients in Japan, 24% had DAH during the observation period, and pre-existing airway involvement was associated with the occurrence of DAH [34]. These findings suggest that caution against lung symptoms is important for Japanese patients with MPA.

While a diagnosis of AAV can be made using physical manifestations as clues, the pathophysiology of AAV can be classified based on autoantibody profiles: PR3-ANCA-positive AAV and MPO-ANCA-positive AAV. PR3-ANCA-positive AAV tends to involve head and neck symptoms more often than MPO-ANCA-positive AAV, whereas MPO-ANCA-positive AAV tends to feature severe renal damage [1], which suggests that PR3-ANCA-positive AAV almost corresponds to GPA, whereas MPO-ANCA-positive AAV almost corresponds to MPA, as included in the above-listed classification criteria [24,25,26,28]. However, regional differences between Europe and Japan regarding the positivity of ANCA have been reported. In a nation-wide survey in Japanese hospitals of patients classified as having AAV based on the criteria proposed by Watts et al. [24], 54.6% of patients with GPA were positive for MPO-ANCA, whereas 45.5% of patients with GPA were positive for PR3-ANCA. In addition, the patients with MPO-ANCA-positive GPA tended to have higher serum creatinine (sCr) levels compared to patients with PR3-ANCA-positive GPA [35]. Dual positivity for PR3-ANCA and MPO-ANCA is rare but has been observed in patients with AAV. In a retrospective study of the Chinese population, similar to Japan insofar that both are in East Asia, 5% of the enrolled patients were classified in the dual-positivity group. In these patients, characteristics of both PR3-ANCA-positive AAV and MPO-ANCA-positive AAV were observed [36]. The positivity rate of MPO-ANCA in EGPA patients ranges from 30 to 35%, which is relatively low compared to that for MPA [21]. The positivity rate of PR3-ANCA in EGPA patients is thought to be quite low: 2.2% in a study conducted by Papo et al. [22]. EGPA is thought to consist of two conditions driven by either eosinophils or ANCA [21]. MPO-ANCA-positive EGPA is thought to be a marker of a disease phenotype; we previously reported that it tended to involve renal damage, such as higher blood urea nitrogen and urine protein levels, as found in a Japanese single-center retrospective study [37].

Although the measurement of serum ANCA is useful for diagnosis, the measurement of serum markers included in a complement system was also reported as useful for predicting the prognosis of AAV in studies on Japanese populations. Fukui et al. analyzed the clinical characteristics of 81 patients with GPA, MPA, and EGPA admitted to Nagasaki University Hospital, located in western Japan, dividing them into a hypocomplementemia group and a normocomplementia group based on the existence of either low complement 3 and complement 4 or total complement activity. A statistically significant higher rate of death was observed in the hypocomplementia group, and a higher mortality rate was also found in a subanalysis excluding EGPA [38]. In addition, in a group with a larger number of patients, low serum C3 levels were associated with renal involvement [39]. Matsuda et al. reported that high serum C4 levels were associated with end-stage kidney disease (ESKD) in another Japanese population of MPA [40]. In a Japanese multicenter study investigating complement cascades in patients with GPA or MPA, properdin levels were inversely correlated with the Birmingham Vasculitis Activity Score, and factor D levels were conversely correlated with the Vasculitis Damage Index [41].

ANCAs are diagnostic biomarkers for AAV, as described above; however, ANCAs might be positive in other rheumatic diseases, such as primary Sjögren’s syndrome [42]. Therefore, precise differential diagnosis is necessary even in patients positive for ANCAs. On the other hand, AAV might be involved in other autoimmune diseases. In a retrospective study on patients with systemic lupus erythematosus treated at a university hospital in the Republic of Korea, a country located in East Asia like Japan, 37.8% of patients diagnosed with lupus nephritis were histologically compatible with AAV based on the 2022 ACR/EULAR criteria [43]. In addition, it has been reported that, from the same institute, 83.3% of patients with polymyositis/dermatomyositis positive for ANCAs were compatible with the overlap of polymyositis/dermatomyositis and AAV [44]. Even though methods of treating AAV have features in common with treatments of the above-listed autoimmune diseases, such as the usage of GCs and immunosuppressants such as CY, RTX, and mycophenolate mofetil (MMF), the treatment for AAV accompanying other autoimmune diseases needs to be appropriately tailored considering the characteristics of each patient and practice guidelines, which vary among countries, as discussed below.

## 3. Treatment of AAV

### 3.1. Treatment of GPA and MPA

In 2022, the EULAR announced an updated version of the recommendations for the treatment of AAV [45]. Then, in 2023, updated guidelines for the management of GPA and MPA, developed as a project of the JMHLW, were published [7,46] as a revised version of the 2017 JMHLW guidelines [8]. GCs, which are fast-acting immunosuppressants, in spite of their accumulating toxicity, were included as part of a remission induction regimen for AAV in the 2022 EULAR recommendations as well as the 2023 JMHLW guidelines. In the 2022 EULAR recommendations, GCs are recommended for use in a remission induction regimen in combination with either CY or RTX for organ- and life-threatening conditions, or in combination with methotrexate (MTX) or MMF for non-organ- and life-threatening conditions. Those doses of prednisolone (PSL) should be tapered to 5 mg/day or equivalent after 4 to 5 months [45]. Combination therapy of GCs and immunosuppressants, such as RTX, CY, MTX, and MMF, was also introduced as a remission induction regimen in the 2023 JMHLW guidelines: GCs should be used with MTX for patients with neither severe organ damage nor renal dysfunction for whom CY and RTX cannot be administered, and GCs should be used with MMF for patients with either severe organ damage or renal dysfunction where CY and RTX cannot be administered. The option of GC monotherapy for patients who have risks such as old age or a renal insufficiency requiring dialysis, or who are at risk of infection, was mentioned in the previous 2017 JMHLW guidelines [8] and remained in the revised 2023 guidelines [7]. In the 2022 EULAR recommendations, 50–75 mg/day of PSL or equivalent GCs is recommended as an initial dose in the remission induction regimen, as well as in the study of [45]. In the PEXIVAS trial [47], which tested the reduction rate of GCs stated in the 2022 EULAR recommendations, the occurrence rate of either ESKD or mortality was non-inferior in the reduced-dose GC group compared to the standard-dose GC group, with the condition that CY or RTX was used in combination with GCs. In the LoVAS trial of the Japanese population, the achievement of remission and the occurrence rate of severe adverse effects were non-inferior in the patient group starting PSL at 0.5 mg/kg/day as an initial dose compared to the group starting PSL at 1.0 mg/kg/day as an initial dose [48,49]. In remission maintenance, it is noted that the GC dose should be reduced and stopped in the 2022 EULAR recommendations [45]. In the 2023 JMHLW guidelines, the combination therapy of GCs with RTX or AZA (in cases where AZA is preferred to RTX) was introduced as a remission maintenance regimen, whereas GC monotherapy for remission maintenance is noted in cases in which GC monotherapy has been carried out for remission induction [7].

CY can be administered either orally or intravenously for patients with AAV. Intravenous CY is superior to oral CY in terms of the reduced risk of infection and a reduction in total administration dose [11]. Oral CY is considered for patients for whom IVCY is not appropriate from the perspective of cost, where there is limited access to infusion, or where there is good adherence to oral intake [50]. Whilst CY is useful for remission induction in the treatment of AAV, the administration of CY may potentially bring about some adverse effects, such as ovarian dysfunction and carcinogenesis [12,13]. RTX is selected in order to avoid these adverse effects; however, CY might be considered for patients with severe renal dysfunction over RTX. In a retrospective study analyzing patients diagnosed with AAV at multiple centers in Japan, CY was found to be better in terms of renal outcomes in the short term in the remission induction period [51]. It was mentioned that CY is preferred for patients with limited access to RTX or severe renal dysfunction due to GN in the Kidney Disease Improving Global Outcomes 2024 Clinical Practice Guideline for the Management of AAV [50]. In the 2023 JMHLW guidelines, oral CY in combination with GCs and plasma exchange (PE) might be considered for patients with the severe renal dysfunction of more than 5.7 mg/dl of sCr [7].

RTX is considered a component of remission induction in parallel with CY and as a remission maintenance regimen in both the 2022 EULAR recommendations [45] and the 2023 JMHLW guidelines [7]. RTX has the advantage of avoiding the adverse effects of CY such as ovarian failure [12,13], and it is mentioned that RTX is preferred for relapsing cases in the 2022 EULAR recommendations [45]. The administration of RTX in combination with a high dose of GCs for remission induction was associated with an elevated risk of severe infection in patients with GPA or MPA who were 75 years old or older in a study conducted by Thietart et al. The investigators hypothesized that the identified risk of infection was mainly attributable to a high dose of GCs because the incidence of serious infections was not elevated when RTX was used for remission maintenance [52]. Considering the results of the PEXIVAS [47] and the LoVAS trials (especially the latter, in which RTX was used in combination with GCs for both patient groups [48,49]), a low initial dose and/or a rapid reduction in GCs is favorable to decrease adverse effects without decreasing therapeutic effects in a remission induction regimen that consists of RTX and GCs.

It is established knowledge that CY and RTX improve the prognosis of patients with AAV; however, the adverse effects discussed above might not be tolerable in patients at high risk such as the elderly. In a study based on a questionnaire sent to medical doctors of multiple disciplines who treated patients with AAV in Japan, non-rheumatologists such as nephrologists and pulmonologists tended to refrain from choosing CY and RTX [53]. This discordance might in part have arisen from differences in patient demographics: Japanese patients with MPA and GPA tend to have a later onset compared to European patients [9,10]. As mentioned above, GC monotherapy might be considered for patients who were considered high-risk cases in the previous 2017 JMHLW guidelines [8].

PE is expected to remove serum ANCAs quickly and is considered for severe forms of AAV. However, in the PEXIVAS trial on patients with ANCA-positive AAV with severe renal dysfunction complications and an estimated glomerular infiltration rate of less than 50 mL/min/1.73 m^2^ or DAH, there were no significant differences in death or ESKD between the patient group with PE and without PE [47]. In addition, the results of a later meta-analysis showed that PE had little to no effect on mortality, while it reduced the 12-month risk of ESKD and was also associated with an increase in serious infections [54]. In the 2022 EULAR recommendations, it is stated that PE might be considered for patients complicated with active GN with sCr levels of more than 300 µmol/L [45]. As mentioned above, in the 2023 JMHLW guidelines, PE in combination with GCs and oral CY might be considered for cases complicated with severe renal dysfunction with significantly elevated sCr levels [7].

The administration of MTX or MMF and concomitant GCs in the remission induction stage is considered for patients who do not have GPA and MPA with organ- or life-threatening conditions in the 2022 EULAR recommendations [45]. The consideration of MTX is similar to that in the 2023 JMLWH guidelines: MTX is considered in cases where CY and RTX cannot be administered where there is no severe organ damage and where renal dysfunction is not severe. In contrast, MMF is considered in cases where CY and RTX cannot be administered and where there is severe organ damage or non-minimal renal dysfunction [7]. MTX and MMF are also mentioned in the 2023 JMHLW guidelines, although the administration of the two immunosuppressants for patients with AAV occurs off-label in Japan at the time of writing this article.

Azathioprine (AZA) is included as an option in remission maintenance alongside a reduction in GCs and stopping avacopan in the 2022 EULAR recommendations [45] and in the 2023 JMHLW guidelines when used in combination with GCs [7]. However, RTX is preferred even in remission maintenance in recent analyses: in RITAZAREM, an unblinded study comparing the effects of RTX and AZA in the remission maintenance of patients with AAV from both European and Japanese facilities who achieved remission in combination with GCs and RTX, RTX was found to be superior to AZA in preventing relapses [55]. AZA should be considered for patients for whom the administration of RTX is not suitable or for pregnant patients [56].

The efficacy of avacopan against GPA and MPA was highlighted in the ADVOCATE study [15]. Avacopan was introduced as an alternative to GCs in remission induction regimens in the 2022 EULAR recommendations [45] and 2023 JMHLW guidelines [7]. Recently, Okubo et al. reported a 78-year-old Japanese patient with GPA complicated with an otitis media lung nodule and nephritis in whom the combination therapy of avacopan and RTX without GCs caused improvements in hearing acuity as well as lung and kidney symptoms [57]. On the other hand, the efficacy of avacopan in patients with severe renal dysfunction with an estimated glomerular filtration rate (eGFR) of less than 15 mL/min/1.73 m^2^ was not analyzed due to the exclusion criteria in the ADVOCATE study, despite the fact that RPGN is one of the main manifestations of AAV, which often results in severe renal dysfunction. Barr et al. reported four cases of AAV with kidney dysfunction with an eGFR of <15 mL/min/1.73 m^2^, all of whom achieved renal recovery with treatment with avacopan in combination with GCs and immunosuppressants such as CY and RTX [58], which suggested that avacopan is useful even in patients with severe renal dysfunction. We recently reported the effectiveness of avacopan with GCs or monotherapy in three patients with MPA, one of whom was on hemodialysis that was terminated with renal recovery after the introduction of avacopan in addition to GCs, which suggests that avacopan in combination with GCs might aid in renal recovery even without the administration of CY or RTX [59]. Taniguchi et al. recently reported an MPA patient without major organ damage in whom azathioprine was terminated due to leukopenia. The concomitant administration of GCs and avacopan led to decreasing titers of MPO-ANCAs, and the authors presumed that GCs and avacopan decreased MPO-ANCAs synergistically [60]. On the contrary, Kubota et al. reported five cases of AAV, all of whom achieved remission with GCs in combination with CY or RTX; however, the addition of avacopan was ineffective for relapse cases, and the authors inferred that the concomitant use of B-cell-depleting immunosuppressants such as RTX is necessary for avacopan [61]. It should be noted that the cases reported by Kubota et al. were not newly diagnosed and had different disease durations (ranging from one to five years); in these patients, the aim of avacopan administration was not remission induction.

A subanalysis of the ADVOCATE study in the Japanese population revealed an efficacy and safety comparable to those for the global population [62]. However, severe liver dysfunction cases associated with avacopan have been predominantly reported in Japan [16,17,18,19,20], which suggests that there are underlying ethnic factors in avacopan-associated liver injury. Kataoka et al. reported a case of MPA in which avacopan was terminated due to elevated biliary enzymes; avacopan could be resumed by gradually increasing the dosage and continuation of ursodeoxycholic acid [16]. Mori et al. analyzed 22 Japanese patients with AAV treated with avacopan, finding nine cases of avacopan-associated liver injury, and older age was identified as a risk factor of severe liver injury [20], which suggests that the later onset in Japanese patients with AAV compared to those in Europe [9,10] might be associated with the high rate of avacopan-associated liver injury in Japan. We recently reported that the rate of the concomitant use of GCs was significantly higher in patients without liver dysfunction than that of patients who developed liver dysfunction in a Japanese population of patients with AAV treated with avacopan [63]. Considering these findings and the cases introduced above [59,60], the concomitant use of GCs might be considered for synergistic effectiveness and decreasing adverse effects on the liver in patients treated with avacopan. The long-term effectiveness and safety of avacopan have not been established: it is stated that avacopan should be stopped after 6 to 12 months in the 2022 EULAR recommendations [45]. The differences in clinical characteristics and practice with respect to GPA and MPA between Europe and Japan discussed above are summarized in Table 1, and the statuses of the immunosuppressants discussed in this article and PE in the 2022 EULAR recommendations [45] and 2023 JMHLW guidelines [7] are summarized in Table 2.

### 3.2. Treatment of EGPA

In the treatment of EGPA, GC is included in the remission induction regimen for both organ-/life-threatening conditions and non-organ-/life-threatening conditions in the 2022 EULAR recommendations as follows: GCs in combination with CY for organ-/life-threatening conditions, and GC monotherapy for non-organ-/life-threatening conditions [45]. Although GC is still the mainstay in treatment for EGPA, the recent introduction of anti-interleukin 5 therapies such as mepolizumab and benralizumab have been revolutionizing clinical practice in EGPA [21]. The efficacy of mepolizumab for EGPA was validated in the MIRRA study, in which the remission induction and GC-sparing effects of mepolizumab were observed in patients with relapsing/refractory EGPA [64]. Later, the non-inferiority of benralizumab to mepolizumab in inducing remission in EGPA was validated in the MANDARA trial [65].

## 4. Conclusions

As a narrative review, this study has limitations as it did not include statistical analyses. However, as discussed in this article, immunosuppressive therapies, including CY, RTX, and avacopan, have improved the prognosis of patients with AAV; however, caution against adverse effects is required, especially for at-risk patients such as the elderly. There are differences in the patient demographics of AAV in terms of ethnicity, which might cause differences in terms of the adverse effects of immunosuppressants. Physicians in contact with patients with AAV should take the characteristics of each patient, such as their age and ethnicity, into consideration when choosing the therapeutic regimen.

## Figures and Tables

**Table 1:** Differences in clinical characteristics and practice regarding GPA and MPA between Europe and Japan.

	Europe	Japan
Classification/diagnostic criteria	The 2022 ACR/EULAR classification criteria for GPA [25] and MPA [26].	The JMHLW criteria for WG (currently GPA) and MPA as diseases designated intractable by the Japanese government [28].
Tendency in clinical phenotypes	GPA is more common.	MPA is relatively common compared to GPA. Later onset compared to European populations. Around half of patients with GPA are positive for MPO-ANCA.
Treatment	The 2022 EULAR recommendations in which RTX is included in remission induction regimens for both organ-/life-threatening conditions, remission induction for non-organ-/life-threatening conditions, and remission maintenance.	Administration rate of CY and RTX is relatively low. Cases of severe liver injury associated with avacopan reported.

ACR, American College of Rheumatology; ANCA, anti-neutrophil cytoplasmic antibody; CY, cyclophosphamide; EULAR, European Alliance of Associations for Rheumatology; GPA, granulomatous with polyangiitis; JMHLW, Japan Research Committee of the Ministry of Health, Labour, and Welfare; MPA, microscopic polyangiitis; RTX, rituximab; and WG, Wegener’s granulomatosis.

**Table 2:** Positionings of immunosuppressants and PE for AAV in the 2022 EULAR recommendations and 2023 JMHLW guidelines.

	2022 EULAR Recommendations [48]		2023 JMHLW Guidelines [7]	
	Remission Induction	Remission Maintenance	Remission Induction	Remission Maintenance
GC	Used in combination with RTX or CY for organ-/life-threatening conditions, MTX or MMF for non-organ-/life-threatening conditions Reduce to 5 mg/day of PSL equivalent after 4 to 5 months	Reduced and stopped	Used in combination with RTX or CY for patients in whom RTX or CY can be administered, in combination with MTX or MMF for patients in whom RTX or CY can be administered Monotherapy is considered for at-risk patients	Used in combination with RTX or AZA (in cases where AZA is preferred to RTX); monotherapy is considered in cases where GC monotherapy was carried out for remission maintenance
CY	For organ-/life-threatening conditions in combination with GCs (or avacopan)	Not included	For patients who can be administered combination therapy with GCs (or avacopan); intravenous administration is preferred to oral	Not included
RTX	For both organ-/life-threatening conditions and non-organ-/life-threatening conditions in combination with GCs (or avacopan)	Included as an option, alongside reducing GCs and stopping avacopan	For patients who can be administered combination therapy with GCs (or avacopan)	Included as an option in combination with GCs
PE	Could be considered for rapidly progressive glomerulonephritis	Not included	Could be considered for severe renal dysfunction in combination with GCs and oral CY	Not included
MTX	In cases with neither organ- nor life-threatening conditions, in combination with GCs (or avacopan)	Included as an option, alongside tapering GCs and stopping avacopan	* In cases where CY and RTX cannot be administered, without severe OD/RD, in combination with GCs	Not included
MMF	In cases with neither organ- nor life-threatening conditions, in combination with GCs (or avacopan)	Not included	* In cases where CY and RTX cannot be administered, with severe OD or non-minimal RD, in combination with GCs	Not included
AZA	Not included	Included as an option, alongside reducing GCs and stopping avacopan	Not included	Included as an option in combination with GCs
Avacopan	Alternative to GCs	Stop after 6 to 12 months	Alternative to GCs	Not included

AAV, anti-neutrophil cytoplasmic antibody-associated vasculitis; AZA, azathioprine; CY, cyclophosphamide; EULAR, European Alliance of Associations for Rheumatology; GC, glucocorticoid; JMHLW, Japanese Ministry of Health, Labor, and Welfare; MMF; mycophenolate mofetil; MTX, methotrexate; OD, organ damage; PE, plasma exchange; PSL, prednisolone; RD, renal dysfunction; RTX, rituximab; and * off-label use in Japan at the time of writing this article.

## Data Availability

No new data were created or analyzed in this study.
